# Supplementary material for: Constructing and sampling partite, 3-uniform hypergraphs with given degree sequence
Source: PLoS One. 2024 May 15;19(5):e0303155. doi: 10.1371/journal.pone.0303155 (PMC11095770; doi:10.1371/journal.pone.0303155)
Supplement: Attachment — Submitted filename: ResponseToReviewers.pdf [file pone.0303155.s001.pdf]

PONE-D-23-27282
Constructing and sampling partite, 3-uniform hypergraphs with given degree sequence
PLOS ONE

Dear Editors,

Thanks for the reviewing process of our manuscript. Below we answer to the referees in detail. We would like to warmly thank the constructive criticism of Referee#2. However, we also would like to mention that we do not agree with some of his/her comments. Most notably, we believe that his/her criticism about the non-correctness of the proof of Theorem 3 does not hold. Still, the issue highlights that the proof of Theorem 3 could be misunderstood. Therefore, we explained it more. We hope that the proof is now easy to understand. Below the original instruction we got are greyed, our answers are highlighted.

On behalf of all authors,
Istvan Miklos

Journal Requirements:

When submitting your revision, we need you to address these additional requirements.

1. Please ensure that your manuscript meets PLOS ONE's style requirements, including those for file naming. The PLOS ONE style templates can be found at https://journals.plos.org/plosone/s/file?id=wjVg/PLOSOne_formatting_sample_main_body.pdf and https://journals.plos.org/plosone/s/file?id=ba62/PLOSOne_formatting_sample_title_authors_affiliations.pdf

Answer: done

2. In your Methods section, please include additional information about your dataset and ensure that you have included a statement specifying whether the collection and analysis method complied with the terms and conditions for the source of the data.

Answer: done

3. Please note that PLOS ONE has specific guidelines on code sharing for submissions in which author-generated code underpins the findings in the manuscript. In these cases, all author-generated code must be made available without restrictions upon publication of the work. Please review our guidelines at https://journals.plos.org/plosone/s/materials-and-software-sharing#loc-sharing-code and ensure that your code is shared in a way that follows best practice and facilitates reproducibility and reuse.

Answer: N/A.

4. Thank you for stating the following in the Acknowledgments Section of your manuscript:
"Our research was supported by the European Union project RRF2.3.1-21-2022-00004 within the framework of the Artificial Intelligence National Laboratory Grant no RRF-2.3.1-21-2022-00004. AH and IM were supported by the European Union project RRF2.3.1-21-2022-00006 within the framework of Health Safety National Laboratory Grant no RRF-2.3.1-21-2022-00006. IM was further supported by NKFIH grant K132696."

We note that you have provided funding information that is not currently declared in your Funding Statement. However, funding information should not appear in the Acknowledgments section or other areas of your manuscript. We will only publish funding information present in the Funding Statement section of the online submission form.
Please remove any funding-related text from the manuscript and let us know how you would like to update your Funding Statement. Currently, your Funding Statement reads as follows:
"Our research was supported by the European Union project RRF2.3.1-21-2022-00004 within the framework of the Artificial Intelligence National Laboratory Grant no RRF-2.3.1-21-2022-00004. AH and IM were supported by the European Union project RRF2.3.1-21-2022-00006 within the framework of Health Safety National Laboratory Grant no RRF-2.3.1-21-2022-00006. IM was further supported by NKFIH grant K132696."

Please include your amended statements within your cover letter; we will change the online submission form on your behalf.

Answer: We removed these funding information from Acknowledgement. The cited funding information is correct. Thanks for handling this on our behalf.

5. Thank you for stating the following financial disclosure:
"Our research was supported by the European Union project RRF2.3.1-21-2022-00004 within the framework of the Artificial Intelligence National Laboratory Grant no RRF-2.3.1-21-2022-00004. AH and IM were supported by the European Union project RRF2.3.1-21-2022-00006 within the framework of Health Safety National Laboratory Grant no RRF-2.3.1-21-2022-00006. IM was further supported by NKFIH grant K132696."

Please state what role the funders took in the study. If the funders had no role, please state: ""The funders had no role in study design, data collection and analysis, decision to publish, or preparation of the manuscript.""
If this statement is not correct you must amend it as needed.
Please include this amended Role of Funder statement in your cover letter; we will change the online submission form on your behalf.

Answer: The funders had no role in study design, data collection and analysis, decision to publish, or preparation of the manuscript.

6. We note that the grant information you provided in the ‘Funding Information’ and ‘Financial Disclosure’ sections do not match.

When you resubmit, please ensure that you provide the correct grant numbers for the awards you received for your study in the ‘Funding Information’ section.

7. Thank you for stating the following in your Competing Interests section:
"No interest"
Please complete your Competing Interests on the online submission form to state any Competing Interests. If you have no competing interests, please state ""The authors have declared that no competing interests exist."", as detailed online in our guide for authors at http://journals.plos.org/plosone/s/submit-now
This information should be included in your cover letter; we will change the online submission form on your behalf.

Answer: The authors have declared that no competing interests exist.

8. We note that you have stated that you will provide repository information for your data at acceptance. Should your manuscript be accepted for publication, we will hold it until you provide the relevant accession numbers or DOIs necessary to access your data. If you wish to make changes to your Data Availability statement, please describe these changes in your cover letter and we will update your Data Availability statement to reflect the information you provide.

Answer: We decided to share our data publicly. DOI already included in the rewritten manuscript.

9. We note that you have included the phrase “data not shown” in your manuscript. Unfortunately, this does not meet our data sharing requirements. PLOS does not permit references to inaccessible data. We require that authors provide all relevant data within the paper, Supporting Information files, or in an acceptable, public repository. Please add a citation to support this phrase or upload the data that corresponds with these findings to a stable repository (such as Figshare or Dryad) and provide and URLs, DOIs, or accession numbers that may be used to access these data. Or, if the data are not a core part of the research being presented in your study, we ask that you remove the phrase that refers to these data.

Answer: We created an additional figure presenting these data.

10. Please include a caption for figure 1.

Answer: we do not understand this comment. Figure 1 did have a caption in the first version of the manuscript, and it still has one.

Additional Editor Comments:

ACADEMIC EDITOR: Please prepare a revised version of the manuscript with all changes colored in red. Moreover, include a point by point answer to all the reviewers comments.

Answer: done.

[Note: HTML markup is below. Please do not edit.]

Reviewers' comments:

Reviewer's Responses to Questions

Comments to the Author

1. Is the manuscript technically sound, and do the data support the conclusions?

The manuscript must describe a technically sound piece of scientific research with data that supports the conclusions. Experiments must have been conducted rigorously, with appropriate controls, replication, and sample sizes. The conclusions must be drawn appropriately based on the data presented.

Reviewer #1: Partly

Reviewer #2: Partly

---

2. Has the statistical analysis been performed appropriately and rigorously?

Reviewer #1: N/A

Reviewer #2: N/A

---

3. Have the authors made all data underlying the findings in their manuscript fully available?

The PLOS Data policy requires authors to make all data underlying the findings described in their manuscript fully available without restriction, with rare exception (please refer to the Data Availability Statement in the manuscript PDF file). The data should be provided as part of the manuscript or its supporting information, or deposited to a public repository. For example, in addition to summary statistics, the data points behind means, medians and variance measures should be available. If there are restrictions on publicly sharing data—e.g. participant privacy or use of data from a third party—those must be specified.

Reviewer #1: No

Reviewer #2: Yes

---

4. Is the manuscript presented in an intelligible fashion and written in standard English?

PLOS ONE does not copyedit accepted manuscripts, so the language in submitted articles must be clear, correct, and unambiguous. Any typographical or grammatical errors should be corrected at revision, so please note any specific errors here.

Reviewer #1: No

Reviewer #2: Yes

---

5. Review Comments to the Author

Please use the space provided to explain your answers to the questions above. You may also include additional comments for the author, including concerns about dual publication, research ethics, or publication ethics. (Please upload your review as an attachment if it exceeds 20,000 characters)

Reviewer #1: The "State of the Art" section needs better documentation. It should provide a concise summary of existing research and compare it more explicitly with the authors' innovative findings. This will enhance the article's clarity and impact.

Answer: We added a paragraph about sampling (non-simple) hypergraphs in the Introduction. We highlighted that earlier approaches worked in the space of non-simple hypergraph realizations of degree sequences (including the rejection sampling method of Dyer *at al.* – they simple rejected any generated non-simple hypergraph).

Reviewer #2: \section*{General comments}

The paper can be divided into two main parts: in the the first one, it is provided the NP-completeness proof of the existence of a partite 3-hypergraph with prescribed degree sequence. Then, a P-time algorithm for the case of third-almost regular degree sequences is defined. The second part of the paper consider a Parallel Tempering method to generate 3-hypergraphs starting from a given one with prescribed degree sequence. The deviation of the degree sequence of the reached hypergraphs from the starting one is considered as energy measure. A χ^2 test is also carried on.

The paper can not be published in the present form, since it suffers from the following major drawbacks:

English is not always appropriate, please check carefully for student's errors that are not appropriate in a scientific paper at all, i.e., plural/singular, third person, past/past-simple/present ...

Section 2 Realizing hypergraph degree sequences has to introduce a standard notation. Refer to the books Graphs and Hypergraphs by Berge and do not deviate from that. B.t.w. use everywhere (hyper)graphical sequences instead of (hyper)graph sequences

Answer: even more „graphic” as this has been the standard notation, see for example, the Deza *et al* papers. We changed it throughout the manuscript.

The NP-completeness proof seems not correct (see detailed comments).

Answer: We believe that the referee is not right here, and our proof is correct. We answered his criticism.

The proofs of Lemma 10 and Thm 11 have to be simplified. I also suggest to write some lines of code and act on them to provide an immediate and clear idea of the involved constructions (see detaied comments).

Answer: We believe that the referee's suggestion of a simplified proof of Lemma 10 is not correct or at least is not mathematically rigorous. We explained it in our answer below.

The Tempering part has to be clarified. The authors have to clearly state what they are going to do at each step and what they obtain.

Answer: We explained the so far raised issues.

Bibliography: Forsini $\rightarrow$ Frosini

Answer: We apologize for the typo, corrected.

By the same authors, it is also appropriate to be aware of the results in
\begin{description}
\item{} Andrea Frosini, Christophe Picouleau, Simone Rinaldi:
New sufficient conditions on the degree sequences of uniform hypergraphs. Theor. Comput. Sci. 868: 97-111 (2021)

\item{} Michela Ascolese, Andrea Frosini, William Lawrence Kocay, Lama Tarsissi: Properties of Unique Degree Sequences of 3-Uniform Hypergraphs. DGMM 2021: 312-324

\item{}Michela Ascolese, Andrea Frosini:
Characterization and Reconstruction of Hypergraphic Pattern Sequences. IWCIA 2022: 301-316

\item{} William Kocay, Pak Ching Li:
On 3-Hypergraphs with Equal Degree Sequences.
Ars Combinatoria 82

\end{description}

Answer: We would like to thank the referee to bring these references to our attention. Those that we found relevant are now cited in the revised manuscript.

Summing up, the problem considered in the paper is interesting and of relevance in the field. It deserves to be studied. The NP completeness proof of Thm 4 needs to

be carefully checked and rewritten; same for the P-time algorithm. Tempering section has to be clarified. The authors left some non trivial open problems.

\section*{Detailed Comments}

\begin{description}
\item{p.1} l.2 one of the most $\rightarrow$ among;

Answer: text changed.

l.3 use parenthesis in degree sequences, i.e., $D=(d_1,d_2\dots,d_n)$

Answer: parentheses added.

l.14 are a generalization of graphs. Simple hypergraphs do exist.

Answer: Text changed according to the suggestion.

l.15 hyperedge, simply edge, ...

Answer: text changed.

\item{p.2}

l.19 is NP-complete

Answer: language corrected.

l.21 algorithms are for the reconstruction of hypergraphs, so they also solve the decision problem

Answer: text changed and extended as: These efficient algorithms can decide if a hypergraph realization exists\added{, and if so, construct a realization in polynomial time}

l.27 who first defined switchings? please refer to Ryser first and then to the switching algebra introduced and studied by Maurice Nivat

Answer: New sentence added: These Markov chains use the \emph{switch operation} introduced by first Havel \cite{Havel}, Hakimi \cite{Hakimi} and \emph{Ryser} and popularized by many others, including Maurice Nivat \cite{Nivat}.

l.28 state that vertices have to be distinct

Answer: new text added:(all vertices must be different).

PAY ATTENTION: here you are dealing with {\em simple} graphs and hypergraphs,

so state that it explicitly. Also state clearly that the consistency and reconstruction problems on non-simple graphs and hypergraphs are easy to solve. As a consequence, it is not possible to use switchings to pass from one simple (hyper)graph to another.

Answer: In this paragraph we deal with simple graphs. It is known that switches are sufficient to transform any realization of a simple graph degree sequence into any another realization of the same degree sequence (via simple graph realizations, of course).

Also the Monte Carlo approach has to face the probability of passing through non simple (hyper)graphs by switchings

Answer: It could be a possibility, however, this is not considered in our manuscript. Instead, we allow that the Markov chain changes the degree sequence of the actual hypergraph. We emphasised it clearly in the rewritten manuscript, and we hope that the description is clear.

l.63 here and all over the paper: remove '' ... '' from words

Answer: Done, except in the sentences „for sake of simplicity, from now by ``hypergraph'' we mean partite \$3\$-uniform hypergraphs" and „Here \$\propto\$ means ``proportional to''", where we feel appropriate.

\item{p.3}

l.121 the definition of hypergraph allows a generic multiset of subset of vertices, so simplicity is not required there.

Answer: Although non-simple hypergraphs also exists, in our manuscript, we would like to consider only simple hypergraphs, and particularly, only 3-partite, 3-uniform simple hypergraphs. Text is changed accordingly.

l.123 the notation \${V}\choose{t}\$ does not exist.

Answer: This notation does exist in the literature of graphs and hypergraphs. Most notably, there is a Frosini *et al* publication that also uses this definition, Andrea Frosini, William L. Kocay, Giulia Palma, Lama Tarsissi: On Null 3-Hypergraphs, https://hal.science/hal-03058519/document

However, we understand that the readership of PLOS ONE might not know this notation, so we added an explanation.

Definition 1 contains more than one definition, and this is not appropriate. Since you do not refer to it in the sequel of the paper it is better to avoid the definition environment and state all the definitions needed in the text. Usually definition environment is used for definitions not in literature, firstly defined in a paper.

Answer: We removed the definition environment and changed the text accordingly. We emphasised that we consider only simple hypergraphs.

Definition 2: a similar operator is in William Kocay, Pak Ching Li: On 3-Hypergraphs with Equal Degree Sequences.
Ars Combinatoria 82. Maybe it can be suitable to provide a symbol for the operator and differentiate it according to the A,B or C sets involved.

Answer: We thank for the referee to draw our attention to this paper. We cited it. We do not feel the need to provide a symbol for the operator.

l.126 broken down $\rightarrow$ split

Answer: done.

l.147 be $\rightarrow$ is

Answer: The sentence here is mandative subjunctive, so we believe that „be” is the correct word.

l.150 adds a non already existing ...

Answer: The next sentence is: We require that neither (a_2,b_1,c_1) nor (a_1,b_2,c_2) be a hyperedge in H before the switch operation. We feel that it exactly describes what the referee would like to read.

\item{pg.4}

in th.4 it is defined the partite 3-uniform hypergraph realization problem, say this otherwise there is no formal definition of the problem.

Answer: Sentence added: The central question is whether or not there is a partite 3-uniform hypergraph with a prescribed degree sequence; we call the corresponding decision problem \textsc{partite 3-uniform hypergraph realization} problem. We will prove that this is a computationally hard problem

Th 6 is useless. It is well known that the problem is NP complete

Answer: We refer to this theorem in the proof of Theorem 3. Therefore, we feel to state it explicitly even if it is well known.

l.124 $V_1,\dots,V_t$ is a partition of V, furthermore, by definition a partition is made by non overlapping subsets

Answer: We do not understand this comment. The definition states that $V_1,\dots,V_t$ is a disjoint partition, which means that the subsets are not overlapping.

\item{pg.5} up to line 190

l.173 define the characteristic function 1_A

Answer: We do not understand this comment. It is already defined in lines 163-164 in the first submission.

ll.187--189 are useless

Answer: This part is deleted from the manuscript.

\item{pg.6}

The NP completeness proof of Thm 4 is inspired by that in [8].

However, the proof seems to have some missing points: in particular the set $E(M)$ defined on line 195 involves the triplets in S such that $w^Tx>0$. Those triplets are not all the triplets with the property. This is a key point in the Deza reduction since it prevents the remaining triplets whose sum is less than or equal to zero from being present.
Here some triplets whose sum is greater than zero and such that 2 or 3 vertices belong to the same set V_i may be missed.
So equation (5) is not assured to be decomposable as $x\in S \cup w^Tx$, a priori, so not always leading to a N-3D-M solution. More in detail: imagine you have V_1 V_2 and V_3 a partition of V and {\em unique} solution of N-3D-M.
In the corresponding hypergraph H it may happen to have two elements, say $v^1_i\in V_1$ and $v^2_j\in V_2$, that you can exchange in all the hyperedges so that the sums remains greater than zero while they the first move from V_1 to V_2 and the latter from V_2 to V_1, preventing the new hypergraph H' from being a solution of N-3D-M.

Please explain carefully this gap in the proof.

Answer: There is no gap here, the referee misunderstood our proof. Although the proof is almost verbatim from the proof of Deza *et al.*, the NP-complete problem used in the reduction has to be changed. Deza *et al*. Used the 3-partition problem to be reduced to the 3-uniform hypergrapic degree sequence problem while we used the nuberical 3-dimensional matching problem to be reduced to the tripartite, 3-uniform hypergraphic degree sequence problem. To picturize:

3-partition problem	≤	3-uni hypergraphic degree seq. realization
numerical 3D matching	≤	tripartite 3-uni hypergraphic degree seq. realization

And now the reduction (denoted by ≤) is almost verbatim from Deza *et al.* Observe that in the 3-partition problem any triplet of weights can be considered. Correspondingly, in a 3-uniform hypergraph any triplet of vertices can form a hyperedge. In the numerical 3-dimensional matching problem, only those triplets are considered that contain exactly one element from each of A, B and C. This is formalized in line 173 in the first version of the manuscript and also explicitly

articulated in lines 175-176. In the tripartite hypergraphic degree sequence realization problem those triplet of vertices can form a hyperedge that contain exactly one element from each of A, B and C. To capture this, S is the set of all indicator vectors of the possible tripartite hyperedges that contains exactly one vertex from each vertex class. In the rewritten manuscript, we explicitly stated this after the definition of S. Therefore, this S actually misses all triplets such that 2 or 3 vertices belong to the same set V_i. However, this is exactly what we need! Indeed, the N-3D-M problem also misses any such triplets! That is why the NP-completeness reduction is correct.

We added text in the manuscript highlighting the fact that although the NP-completeness reduction is almost verbatim from Deza *et al.*, the NP-complete problem in the reduction has to be changed.

\item{pg.7}

Lemma 10: there is no need of a so long, complicated and hard to follow proof. Consider the integer matrix whose row sums are $R=(d(c_1), \dots, d(c_n))$ and column sums $C=(d(b_1), \dots, d(b_m))$, with $d(x_y)$ the degree of the vertex $x_y\in X$ in H (consider both vectors arranged in decreasing order). Since H' is B-balanced w.r.t. (A,B), then the multiedges can be arranged so that C' (the correspondent of C w.r.t. H') is almost regular (i.e. the degrees of the elements of B in H' are ${\sum_{i\in |A|}d(a_i)} / m)$ or ${\sum_{i\in |A|}d(a_i)} / m)-1$). Since $B'\leq B$ w.r.t. the dominance order, then a realization M of the matrix whose row and column sums are R and C implies an integer realization M' of the matrix whose row and column sums are R and C' (see [30] and successive results generalized to integer matrices). The elements in the column related to b_i of M' provides the parallel edges of the (B,C)-projection of H' related to b_i and so they can be connected to the elements of A incident to b_i in circular sequencing, i.e. from a_1 to $a_{|A|}$ and starting again, so preserving the balancedness of their degrees in the assignment and avoiding parallel edges (this holds since each integer in the column of b_i is less than $|A|$). So, you get the hyperedges of H'.

Answer: It is completely unclear what the referee would like to say here, and we are afraid that it cannot be considered as a mathematically rigorous proof. For example, the third sentence of this claimed proof starts that „Since H' is B-balanced”. In the first two sentence, it is not proved that H’ exists. However, Lemma 10 states the existence of H’, and so, that should be proved. If in the proof it is assumed that H’ exists (the referee states properties of it), then it will be a circular proof, would not it?

We do not understand the notation B’\leq B. For us, B and B’ are vertex sets. Even if the referee would like to refer to their degree sequences it makes no sense since H and H’ are the realizations of the same degree sequences, therefore the degree sequence of B and B’ are the same. The only thing we can imagine that the referee is talking about some projections (see definition 8 [7 in the rewritten manuscript]) but then it is not stated which projection we are talking about.

Nevertheless, we do not see how this claimed proof proves that H’ can be obtained from H by switch operations. Our proof explicitly constructs such a series of switch operations.

Actually, proving the existence of H’ is indeed quite trivial, we agree with the referee in that issue. However, proving that H can be transformed into H’ with a series of switch operations is significantly harder. That is why we need a proof that is more complicated than the proof merely proving the existence of H’.

We also have to remark that the proof of Kocai and Li that the referee drew our attention is very likely can be extended to tripartite 3-uniform hypergraphs. We now mention it in the manuscript. However, we do not have access to that paper, and therefore, we came up with the current proof of Lemma 10 (lemma 9 in the revised manuscript).

\item{pg.10}

l.337 uniform the notation := and = all over the paper

Answer: done

Lemma 12 is correct. However, the idea is the same as Lemma 10 stated above, so I suggest to define a standard B-balanced realization as in Lemma 10 above and use it to show Lemma 12 and Thm 13 since both H_1 and H_2 can be reduced to it by switches only.

Answer: We kind of feel what the referee would like to say here. We can see that it is easy to create a canonical (or standard) B-balanced realization. However, we do not see if transforming an arbitrary realization to a standard B-balanced realization is easier than transforming the same arbitrary realization into *some* B-balanced realization.

\item{pg.11}

Thm 13 can be included in Lemma 12

Answer: It indeed could be included, but we feel it more digestible to split the statements. The proof of Theorem 13 is quite short due to the thorough work in the proofs of the earlier lemmas. If a reader does not want to understand the technicalities of the previous lemmas then s/he still could get the overall picture from the proof of Theorem 13.

\item{pg.12}

Thm 14 is fine. Maybe few lines of code could help the comprehension.

Answer: We assume that the referee is talking about the part of the proof of thm 14 where we prove the existence of certain hinge-flips. The understanding of which part

of it could be improved by a pseudo-code? Given two arrays of integers D* and D', finding the largest index for which d*_i> d_i? The smallest index for which d*_j< d'_j? Finding a hinge-flip is not explicitly constructive, the existence of such a hinge-flip is a triviality based on the Pigeonhole Principle. Finding such a hinge-flip can be done by exhaustive search by looking at the list of hyperedges incident with the indicated vertices, and obviously can be found in polynomial time. And there is nothing else in the proof of this part…

l.466-468 this sentence has to be clarified! If you consider non simple hypergraphs the answer is yes, there does exist a sequence of switches that allow to pass from any realization of D to any other (Ryser switching theory). On the other hand, if you consider simple 3-hypergraphs only, the NP-completeness proof in [8] prevent the result, i.e. in general, there do not always exist a switches sequences that lead from one solution to another going through simple hypergraphs only.

Explain better also the difference that you mention between graphs and hypergraphs about irreducibility.

Answer: We clarify clearly at the beginning of the manuscript that we consider only simple hypergraphs. We do not agree with the referee why an NP-completeness proof of existence of a realization would indicate the non-existence of a series of switches transforming any realization into another. Nevertheless, here the referee contradicts to Frosini *et al* (https://hal.science/hal-03058519/document). In that paper, it is claimed that N_5 and N_6 in Fig 1 of that paper (of which N_6 is exactly what is a switch in our manuscript, and N_5 is another version of a switch that cannot exist in a tripartite 3-uniform hypergraph but can exists in 3-uniform hypergraphs) are sufficient to transform one realization of a 3-uniform degree sequence into any another realization. So what is the truth? Kocay is also an author of the cited paper which refers to Kocay and Li mentioned above. Unfortunately, we do not have access to the Kocay and Li paper, so we cannot check it.

Still, we significantly rewritten the text here. For us, the cited Frosini paper suggest that actually switches might be irreducible on the (simple) realizations of 3-uniform hypergraphic degree sequences. However, this does not influence in any way our manuscript. Even if switches happen to be reversible on 3-uniform hypergraphs, the Parallel Tempering idea still improves the mixing of our Markov chain.

In the rewitten paragraph, the difference between graphs and hypergraphs are explained better.

\item{pg. 13}

l.487-494 state explicitly that those perturbations maintain the simplicity of the hypergraph. In fact, if not, you can use switches only to pass from an hypergraph to another keeping the degree sequence.

Answer: We emphasized that we consider only simple hypergraphs.

\item{pg. 14}

l.508 what is the purpose of the set X here?

Answer: Indeed, it is an unnecessary notation here, text is changed. We did keep this notation in the description of hinge-flips. Here X should be considered as it was a running index, just it is not an index but a variable that can take a value A, B or C, where A, B and C are the three vertex classes of the hypergraph.

\end{description}

The remaining part of the paper is subdued to the clarification of this info.

---

6. PLOS authors have the option to publish the peer review history of their article (what does this mean?). If published, this will include your full peer review and any attached files.

If you choose “no”, your identity will remain anonymous but your review may still be made public.

Do you want your identity to be public for this peer review? For information about this choice, including consent withdrawal, please see our Privacy Policy.

Reviewer #1: No

Reviewer #2: No
