# Supplementary material for: Constructing and sampling partite, 3-uniform hypergraphs with given degree sequence
Source: PLoS One. 2024 May 15;19(5):e0303155. doi: 10.1371/journal.pone.0303155 (PMC11095770; doi:10.1371/journal.pone.0303155)
Supplement: Attachment — Submitted filename: ResponseToReviewers.pdf [file pone.0303155.s002.pdf]

PONE-D-23-27282
Constructing and sampling partite, 3-uniform hypergraphs with given degree sequence
PLOS ONE

Dear Editors,

Thanks for the reviewing process of our manuscript. Below we answer to the referees in detail. We would like to warmly thank the constructive suggestion of Referee#2.

On behalf of all authors,
Istvan Miklos

- there have been a misunderstanding in the following result: consider simple 3-hypergraphs only. You can easily costruct an example (see the 3-hypergraphs solutions of the 3-partition problem instance A=(1 1 1 2 3 4) constructed according to the procedure in the Deza NP-completeness proof) of a degree sequence that is shared by two only non isomorphic 3-hypergraphs H1 and H2 such that you can not move from H1 to H2 by one single elementary switch.

Answer: We do not think that 6 weights/vertices is enough for a counterexample. With 6 weights, a solution to a 3-partitioning problem instance consists of 2 3-sets, and if there are 2 realizations of the corresponding degree sequence, the symmetric difference of these realizations contains 4 hyperedges, related to a switch operation.

However, the suggested strategy is excellent: find a 3-partition problem instance that has exactly 2 solutions, and these two solutions should differ in at least 6 triplets. It is easy to see that the 3-partition problem instance A = {1,3,4,5,6,7,8,9,11} has exactly 2 solutions. The corresponding degree sequence is D = (4,8,10,12,13,16,17,19,24). It has exactly 2 realizations, and their symmetric difference contains 6 hyperedges. Similarly, the numerical 3-dimensional matching problem instance {1,2,3},{1,2,3},{1,2,3} has 2 solutions. The corresponding tripartite degree sequence is D = (2,4,7), (2,4,7), (2,4,7). It has two realizations; their symmetric difference contains 6 hyperedges. We added these examples to the manuscript.

- the number of mispelled Forsini instead of Frosini name in the bibliography increases from 1 to 2. I expected it reduces to 0 according to your statement.

Answer: we apologize for overlooking some of the cases. Now all misspelled cases are corrected.
